# Identification of a novel anoikis‐related gene signature to predict prognosis and tumor microenvironment in lung adenocarcinoma

**DOI:** 10.1111/1759-7714.14766

**Published:** 2022-12-11

**Authors:** Xiayao Diao, Chao Guo, Shanqing Li

**Affiliations:** ^1^ Department of Thoracic Surgery Peking Union Medical College Hospital, Chinese Academy of Medical Sciences and Peking Union Medical College Beijing China

**Keywords:** anoikis, lung adenocarcinoma, prognostic signature, tumor microenvironment

## Abstract

**Background:**

Lung adenocarcinoma (LUAD) is the most prevalent histotype of non‐small cell lung cancer. Anoikis, an alternative form of programmed cell death, plays a pivotal role in cancer invasion and metastasis, preventing the detached cancer cells from readhering to other substrates for abnormal proliferation. The aim of this study was to conduct a comprehensive analyses of the prognostic implications of anoikis‐related genes (ARGs) in LUAD.

**Methods:**

ARGs were selected from The Cancer Genome Atlas (TCGA) database and Genecards dataset using differential expression analysis. The signature incorporating ARGs was identified using univariate Cox regression analysis and LASSO regression analysis. Furthermore, a nomogram containing the signature and clinical information was developed through univariate and multivariate Cox regression analysis. Kaplan–Meier survival analysis and receiver operating characteristic (ROC) curves were applied to evaluate the predictive validity of these risk models. Finally, functional analysis of the selected ARGs in signature and analysis of immune landscape were also conducted.

**Results:**

A 16‐gene signature was integrated to stratify LUAD patients into different survival risk groups. The prognostic risk score generated from the signature and TNM stage were identified as independent prognostic factors and utilized to develop a nomogram. Both the signature and the nomogram showed satisfactory prediction performance in predicting overall survival (OS) of LUAD patients. The ARGs were enriched in several biological functions and signaling pathways. Finally, differences of immune landscape were investigated among the high‐ and low‐risk groups stratified by the signature.

**Conclusions:**

This study revealed potential relationships between ARGs and prognosis of LUAD. The prognostic predictors identified in present study could be utilized as potential biomarkers for clinical applications.

## Graphical abstract

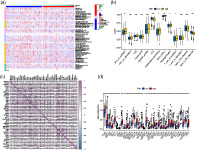

## INTRODUCTION

Lung cancer ranks the second most common and first deadliest malignant tumor worldwide.^1^ Lung cancer also has the highest incidence and mortality in China, and the 5‐year survival rate remains between 10%–15%.^2^ Approximately 80%–85% of lung cancer cases are non‐small cell lung cancer (NSCLC), and lung adenocarcinoma (LUAD) is the most prevalent pathological type.^3^ Although certain breakthroughs have been achieved in the diagnostic and therapeutic technique, the long‐term survival rate of LUAD patients is still poor.^4^ Therefore, identification of the novel and effective biomarkers or therapeutic targets for patients with LUAD is urgently required.

Anoikis is a specific form of programmed apoptosis caused by the detachment of cell–cell or cell‐extracellular matrix (ECM), which plays a pivotal role in maintaining tissue homeostasis by preventing dislodged cells from readhering to other substrates for abnormal proliferation.^5^ The emergence of anoikis resistance could help detached cells abscond programmed cell death procedure, thus preventing them from surviving elsewhere.^6^ Anoikis has therefore been identified as a vital factor in tumor progression. Currently, impaired ability to trigger programmed apoptosis has become a hallmark of cancer, resulting in tumor development and progression.

In recent years, an increasing number of anoikis‐related genes (ARGs) have been identified in various tumors. Ye et al. confirmed that nuclear MYH9 promotes *CTNNB1* transcription, which conferred resistance to anoikis in gastric cancer.^7^ A previous study revealed that the formation of a protein complex containing Bim‐EL, LC8, and Beclin‐1, could contribute to anoikis evasion in inflammatory breast cancer.^8^ Moreover, GDH1‐mediated metabolic reprogramming of glutaminolysis has been verified to promote anoikis resistance and tumor metastasis in LKB1‐deficient lung cancer.^9^ In another study by D'Amato et al., TDO2 was found as an attractive factor to facilitate anoikis resistance in triple‐negative breast cancer.^10^ In addition, ARGs have been proven to be associated with prognosis in several tumors.^11^, ^12^, ^13^ However, the comprehensive analyses of the implications of anoikis in LUAD are still scarce. Therefore, identification of the predominant genes related to anoikis with prognostic significance in LUAD is vital.

In the present study, we first explored the differential expression of ARGs in LUAD from a public database. Second, a prognostic multigene signature based on differentially expressed ARGs was constructed to evaluate the prognosis of LUAD patients. Furthermore, we performed functional analysis to explore the association between ARGs and the tumor microenvironment. This study revealed the prognostic value of ARGs in LUAD and constructed a prognostic model to predict prognosis in LUAD patients.

## METHODS

### Identification of anoikis‐related genes in LUAD

The RNA sequencing data and corresponding clinical information of 535 LUAD patients, including 535 LUAD samples and 59 noncancerous adjacent samples (NATs), were extracted from the TCGA database (https://portal.gdc.cancer.gov). A total of 338 ARGs were downloaded from the GeneCards database (https://www.genecards.org/), and genes with a relevance score >1.0 included in the present study (Table S1). Then, the differential analysis of the ARGs between LUAD and NATs was conducted using the “limma” R package after obtaining intersection from the above two datasets. The *p*‐value was adjusted with the false discovery rate (FDR). FDR <0.05 and |log2 (FC)| ≥ 1 was considered statistically significant. A heatmap and volcano plot were generated using the “pheatmap” R package to visualize differentially expressed ARGs in LUAD.

### Development of the prognostic anoikis‐related genes signature in LUAD

Patients with follow‐up of more than 30 days were included in following study. Patients were randomly divided into the training and validation sets at a 7:3 ratio. Univariate Cox analysis for association with overall survival (OS) was conducted to identify the prognostic ARGs with a criterion of *p* < 0.05. A least absolute shrinkage and selection operator (LASSO) regression algorithm was performed to further screen genes tightly related to OS in LUAD using the “glmnet” package, and 10‐fold cross‐validation was used to select candidate ARGs and identify the penalty parameter (λ), corresponding to the minimum value of partial likelihood deviance.^14^ Finally, a prognostic signature was constructed based on the gene expression levels and their respective coefficients.

Additionally, we used the median risk score as a cutoff to stratify all eligible patients into high‐ and low‐risk groups. Survival differences between high‐ and low‐risk patients were illustrated using Kaplan–Meier survival analysis using the “survival” and “survminer” R packages. The time‐dependent receiver operating characteristic (ROC) curve analysis was conducted to evaluate the discrimination performance of the signature using “timeROC” R package. The consistent formula and cutoff point (the median of risk scores in the training set) were also utilized to generate the risk score of each patient in validation set and divided them into high‐ and low‐risk group. We then applied the validation and entire LUAD cohorts to assess the above findings.

### Construction and evaluation of the nomogram containing anoikis‐related genes signature

In the entire LUAD cohort, univariate and multivariate Cox regression analyses were conducted to identify independent prognostic predictors from the risk score calculated from the signature and several clinicopathological factors, including age, gender, TNM stage, T stage, N stage, and M stage. A nomogram based on identified independent variable factors was established using the “rms” R package. Then, the discrimination and calibration of the nomogram was assessed in the entire LUAD dataset by the ROC and calibration curves. Subsequently, the decision curve analysis (DCA) was used to evaluate the clinical usefulness of the nomogram.

### Functional analysis

To explore the potential biological mechanism and functions of the selected ARGs in signature, we performed gene ontology (GO) and Kyoto Encyclopedia of Genes and Genomes (KEGG) enrichment analyses for the ARGs. The functions or pathways with *p*‐value <0.05 were defined as significantly enriched. Functional enrichment analysis was performed using the “clusterProfiler” R package. Furthermore, to explore the relationship between risk scores calculated from these ARGs and the immune cell abundance, immune function, and immune checkpoints, the immune landscape of LUAD was compared between two risk cohorts using the “limma” R package.

### Statistical analysis

Statistical analyses of this study were performed using the R software, version 4.0.2 (https://www.r-project.org). For comparison of the differences of categorical and continuous variables, the Chi‐square and Student's *t*‐tests were utilized, respectively. The OS between different risk groups was compared using the Kaplan–Meier analysis with the log‐rank test. Univariate Cox regression analysis, multivariate Cox regression analysis and LASSO regression analysis were conducted to identify optimal prognostic predictors of OS. For each analysis in the present study, statistical significance was set at *p* < 0.05.

## RESULTS

### Identification of anoikis‐related genes

**FIGURE 1:**
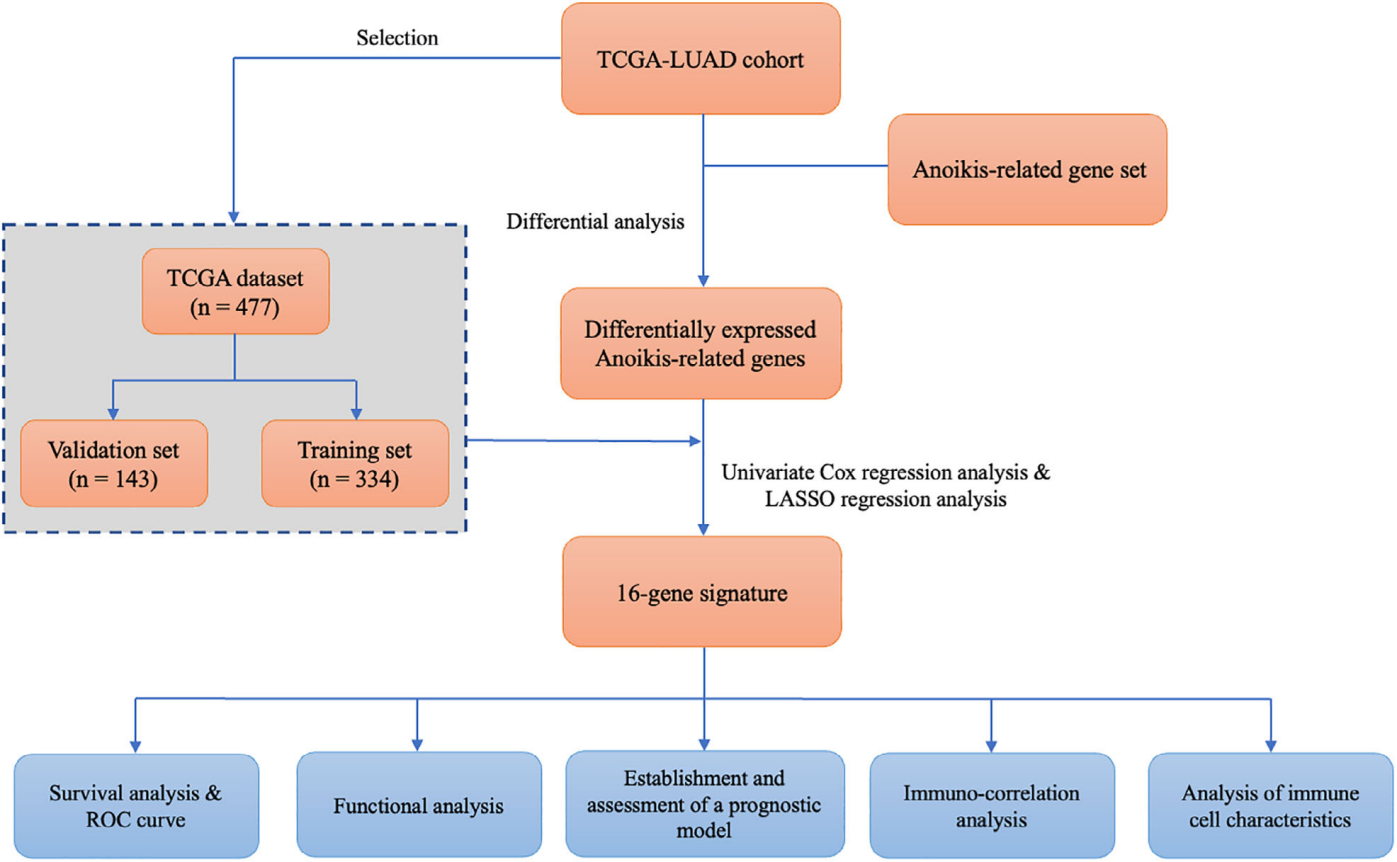
The design and flow chart of this study

**FIGURE 2:**
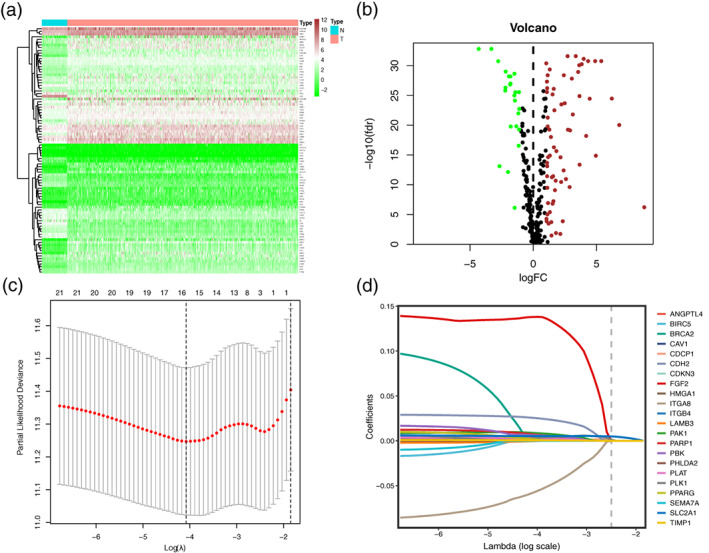
Identification of the anoikis‐related genes (ARGs) in LUAD. (a) Heatmap and (b) volcano plot of differentially expressed ARGs in lung adenocarcinoma. (c, d) The least absolute shrinkage and selection operator (LASSO) coefficient profiles of the 22 genes at different tuning parameters (λ), 10‐fold cross‐validation to filtrate candidate ARGs in LASSO regression analysis

The flowchart of this study is illustrated in Figure 1. We obtained RNA transcriptome data and corresponding clinical data of 535 LUAD patients in TCGA database. Among 338 ARGs identified from the Genecards dataset, 91 differentially expressed ARGs were significant between tumor and NATs, including 67 upregulated and 24 downregulated in tumors (Figure 2a,b).

### Development and validation of the anoikis‐related genes signature

**FIGURE 3:**
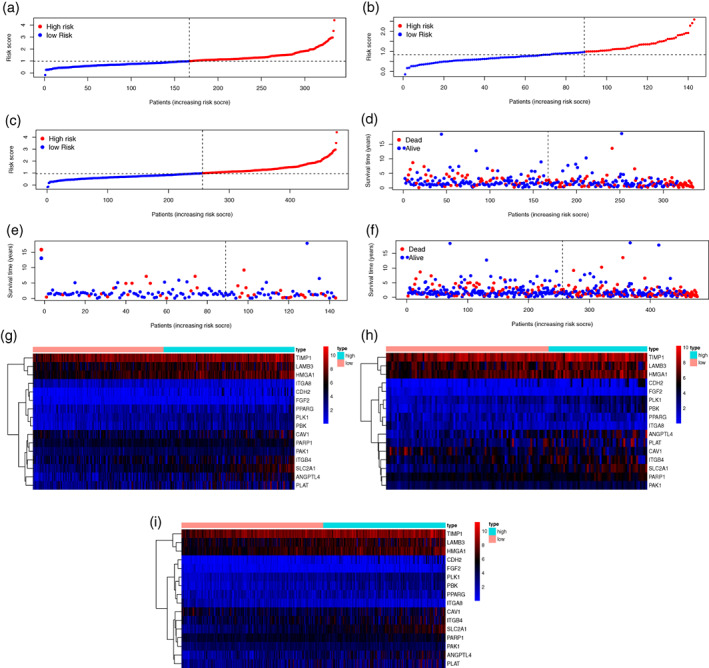
Risk score signature establishment based on anoikis‐related genes (ARGs). (a–c) Distribution of low‐ and high‐risk group lung adenocarcinoma patients divided by the signature in the training, validation, and entire set, respectively. (d–f) Survival statuses of patients in different risk groups grouped by the signature in the training, validation, and entire set, respectively. (g–i) Expression of the selected ARGs in the training, validation, and entire set, respectively

**FIGURE 4:**
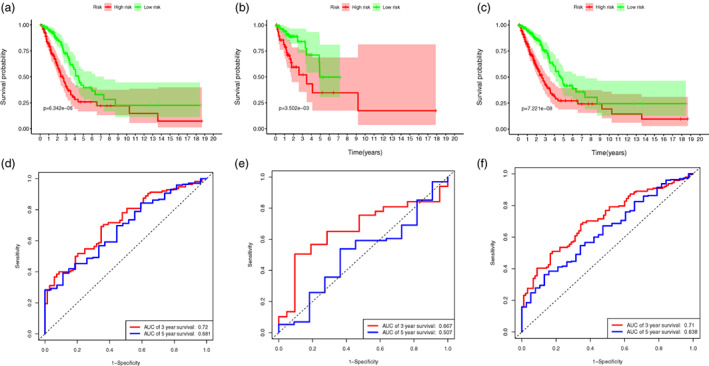
Assessments of the anoikis‐related genes signature. (a–c) Kaplan–Meier survival analysis curves of the high‐ and low‐risk groups divided by the signature in the training, validation, and entire set, respectively. (d–f) Time‐dependent ROC curves at 3‐ and 5‐year follow‐up in the training, validation, and entire set, respectively

To establish the signature for predicting the survival status of LUAD patients, a total of 477 patients, who met the inclusion and exclusion criteria, were randomly grouped into a training set (334 patients) and a validation set (143 patients) at a 7:3 ratio. In the training set, we performed univariate Cox regression on the differentially expressed ARGs and identified 22 genes associated with prognosis. Sixteen optimal prognostic ARGs were further selected by LASSO analysis (Figure 2c,d). Finally, a risk score model including the selected ARGs was developed in the training set: $\text{Risk score}=\sum_{i=1}^{n}\text{coefficients}*\text{Expression of ARGs}\left(i\right)$ (Table 1). Patients in the training, validation, and entire sets were stratified into two groups; a high‐ and a low‐risk group, based on the median risk score calculated from the above formula in the training set (Figure 3a–c). Figure 3d–f showed that increasing risk score was positively correlated with accumulated patients with poor survival status. The expression levels of the ARGs in the signature are illustrated in Figure 3g–i. As shown in the Kaplan–Meier survival analysis, the OS of the high‐risk group was considerably shorter compared to the low‐risk group in all three sets (Figure 4a–c). ROC curve analyses demonstrated that the ARGs signature also possessed a satisfactory discriminative performance (Figure 4d–f).

**TABLE 1:** The prognostic significance of the 16‐gene signature

Anoikis‐related gene	Coefficient	Hazard ratio
CDH2	0.0256	1.0353
PARP1	0.0073	1.0134
LAMB3	0.0003	1.0020
ANGPTL4	0.0030	1.0069
PPARG	0.0018	1.0341
PAK1	0.0089	1.0261
CAV1	0.0012	1.0025
PLAT	0.0026	1.0033
FGF2	0.1380	1.3377
ITGA8	−0.0577	0.8844
ITGB4	0.0023	1.0071
HMGA1	0.0003	1.0019
SLC2A1	0.0052	1.0084
PLK1	0.0027	1.0493
TIMP1	0.0005	1.0009
PBK	0.0064	1.0390

### Construction and evaluation of the nomogram incorporating the anoikis‐related gene signature

**FIGURE 5:**
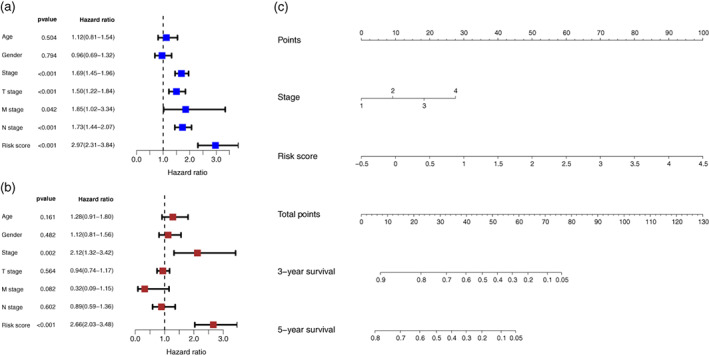
Construction of the nomogram based on the risk scores calculated from the signature. (a, b) The variables selected by univariate and multivariate Cox analyses. (c) A prognostic nomogram was developed to predict the 3‐ and 5‐year survival for lung adenocarcinoma patients

**FIGURE 6:**
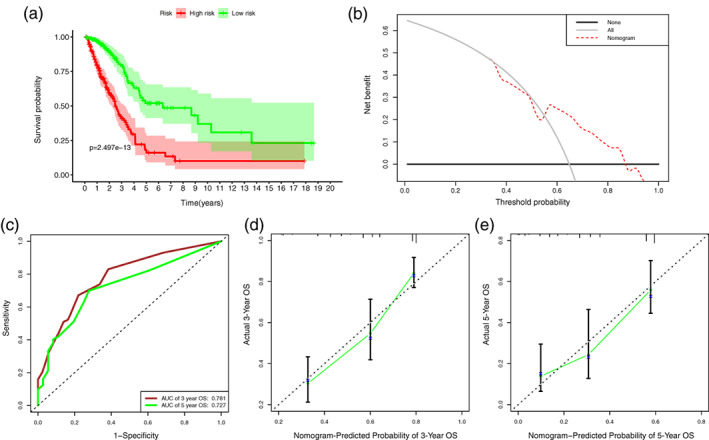
Evaluation of the performance of the nomogram. (a) Kaplan–Meier survival analysis curves of the high‐ and low‐risk groups divided by the nomogram. (b) Decision curve analysis assessing the clinical usefulness of the nomogram for lung adenocarcinoma patients. (c) Time‐dependent receiver‐operating curves at 3‐ and 5‐year follow‐up. Calibration curves show the calibration power of the nomogram for (d) 3‐ and (e) 5‐years

In the entire LUAD set, it was observed that TNM stage, T stage and risk score were closely correlated to OS in the univariate Cox regression. Multivariate Cox analysis further identified that risk score and TNM stage were independent prognostic factors (Figure 5a,b). All variables which were significant in the multivariate Cox regression analysis were enrolled in the predictive model to predict 3‐ and 5‐year OS for LUAD patients (Figure 5c). According to the risk scores generated from the nomogram, LUAD patients in the entire set were grouped into different risk groups by the median value of the risk score. Figure 6a demonstrated that patients in the high‐risk group possessed significantly inferior prognoses compared to those in the low‐risk group. The DCA analysis illustrated that more than half of the red dashed curve was in the area above the gray and the black solid lines, demonstrating a higher net benefit could be obtained through using the nomogram to reach a decision (Figure 6b). In addition, we utilized ROC curves to assess the predictive performance of 3‐ and 5‐year OS of this prognostic model and obtained AUCs of 0.781 and 0.727, respectively (Figure 6c). Furthermore, a good agreement between the nomogram prediction and actual observation was shown in the calibration curves (Figure 6d,e). Consequently, promising predictive value was indicated for this prognostic integrated nomogram.

### Functional analysis

**FIGURE 7:**
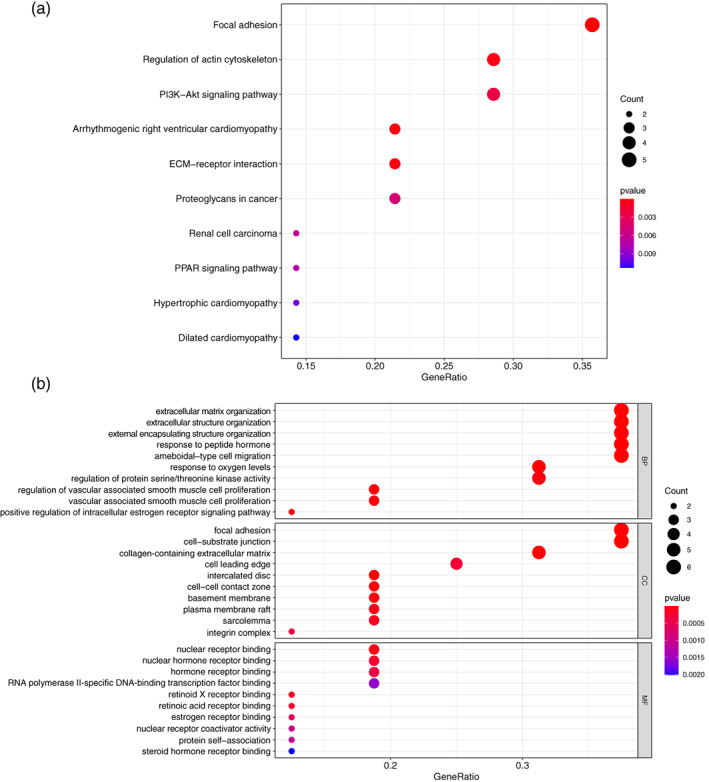
Functional enrichment analysis of anoikis‐related genes (ARGs) in the signature. The (a) gene ontology and (b) Kyoto Encyclopedia of Genes and Genomes terms enriched by the selected ARGs in the gene signature

To explore the underlying mechanisms and functions, we performed functional enrichment analyses to reveal the potential biological functions associated with the ARGs involved in signature. As illustrated in Figure 7a, the KEGG enrichment analysis demonstrated that the correlated genes were mainly clustered in several terms, such as focal adhesion, regulation of actin cytoskeleton, PI3K‐Akt signaling pathway, ECM‐receptor interaction, PPAR signaling pathway and so on. At the same time, GO functional terms of correlated genes were significantly enriched in several biological processes or molecular functions, such as extracellular matrix organization, extracellular structure organization, external encapsulating structure organization, focal adhesion, cell‐substrate junction, nuclear receptor binding, and nuclear hormone receptor binding (Figure 7b). Taken together, these results suggested that the ARGs may be mainly associated with LUAD migration and metastasis.

**FIGURE 8:**
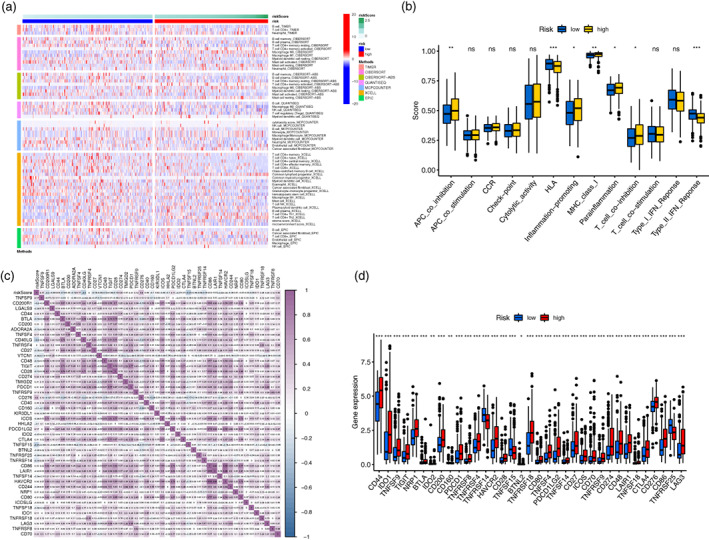
Analysis of immune landscape. (a) Heatmap for discrepancies in abundance of tumor‐infiltrating immune cells among two risk groups stratified by the signature. (b) Immune function analysis among the high‐ and low‐risk groups. (c) Association of immune checkpoints with the risk score generated from the signature. (d) Differences of the expression of immune checkpoints between the two risk groups. *p < 0.05; **p < 0.01; ***p < 0.001

As shown in Figure 8a, the abundances of activated memory CD4+ T cells, M0 macrophages, neutrophils, and resting mast cells were markedly enriched in the high‐risk compared to the low‐risk group. Immune functions including APC coinhibition, MHC class I, inflammation‐promoting, parainflammation, and T cell coinhibition were relatively active in the high‐risk group, while HLA function and type II IFN response were more active in the low‐risk group (Figure 8b). In the analysis of immune checkpoints, the results demonstrated a high expression of immunosuppressive receptors, such as CTLA4, PD‐1, LAG3, BTLA, and TIGIT, and immunosuppressive ligands, such as PD‐L1, PD‐L2 and TNFSF14, in the high‐risk compared to the low‐risk group (Figure 8c,d).

## DISCUSSION

Lung cancer is one of the malignancies that imperils human health, with 2.2 million new cases of lung cancer and 1.8 million deaths reported worldwide in 2020.^1^ The clinical outcomes of lung cancer patients are associated with risk factors and tumor stage at the time of diagnosis. Due to the lack of specific symptoms, more than two‐thirds of patients are diagnosed at advanced stages, leading to an unsatisfactory prognosis.^15^ LUAD is among the most common kind of lung cancer.^16^ Thus, investigatation of the novel molecular markers related to diagnosis and individualized prognostic prediction for patients with LUAD is urgently required. Anoikis, a physiological programmed apoptosis, is essential for maintaining tissue homeostasis through eliminating detached cells to prevent cell adherence in an unfavorable place.^17^ Anoikis evasion has been identified as a significant factor facilitating tumor invasion and metastasis.^18^ Therefore, ARGs have attracted plenty of attention for their promising prognostic value in LUAD.

In this study, ARGs and expression profile from a public sequence dataset were utilized to identify the differentially expressed ARGs related to LUAD. Additionally, a signature based on 16 ARGs and a predictive model incorporating this signature were developed, with satisfactory discriminatory accuracy for predicting OS of LUAD. The potential mechanism of these ARGs were diverse; for example, CDH2, also known as N‐cadherin, could confer anoikis resistance in esophageal carcinoma with high intracellular PKCK2 activity.^19^ PARP1 enhances LUAD metastasis through promoting anoikis resistance.^20^ LAMB3, a regulator of the integrin‐FAK‐Src pathway, promotes cancer progression by anoikis resistance.^21^ ANGPTL4 has also been revealed to enhance anoikis resistance and tumor metastasis via the upregulation of MMP‐1 expression in head and neck squamous cell carcinoma.^22^ In hepatocellular carcinoma, anoikis resistance of hepatoma cells has been determined to require PAK1 activity,^23^ and CAV1 could also confer resistance of hepatoma cells to anoikis by activating the IGF‐1 pathway.^24^ Moreover, ITGB4 promotes metastasis of hepatocellular carcinoma by conferring anchorage independence through EGFR‐dependent FAK‐AKT activation.^25^ HMGA1 promotes anoikis resistance through a PI3K/Akt‐dependent mechanism in pancreatic adenocarcinoma.^26^ PLK1 protects esophageal carcinoma cells from anoikis through regulating β‐catenin protein levels.^27^ Additionally, TIMP1, integrating with CD63 and β1‐integrins as a complex, confers anoikis resistance by activating PI3K signaling pathway in melanoma.^28^

To investigate the potential functions or mechanisms of the gene signature, functional enrichment analysis was performed. KEGG enrichment analysis demonstrated that the selected genes were mainly enriched in focal adhesion, regulation of actin cytoskeleton, PI3K‐Akt signaling pathway, ECM‐receptor interaction and so on. First, the majority of ARGs are associated with focal adhesion, which are cell‐matrix contacts that mature from nascent focal complexes containing integrins and a few other molecules.^29^ Focal adhesion plays major roles in cell invasion, migration and metastasis in non‐small cell lung cancer.^30^ Moreover, actin cytoskeleton plays a key role in many aspects of human physiological or pathological processes, including cancer metastasis. These complex processes involve the coordinated formation of multiple actin‐based structures.^31^ The PI3K‐AKT pathway regulates cell growth and proliferation and is often dysregulated in multiple cancers due to genetic and epigenetic alteration. Activation of this pathway in NSCLC leads to disease progression which is associated with a poor prognosis.^32^ Furthermore, remodeling of ECM, such as expression changes in collagens, proteases and integrins in stroma, provides a permissive environment for tumor progression.^33^ In addition, GO analysis of these selected ARGs also showed that genes were mainly enriched in terms related to cell‐matrix adhesions, cell motility and ECM alteration, which are correlated with the biological process of anoikis.

Furthermore, we found that LUAD patients in the high‐risk group had higher infiltration of tumor‐infiltrating immune cells, such as M0 macrophages, neutrophils, resting mast cells, and activated memory CD4+ T cells, than patients in the low‐risk group. A previous study revealed that an increased number of M0 macrophages was associated with poor prognosis in LUAD at an early clinical stage.^34^ Although memory CD4+ T cells have been proven to possess the ability of tumor suppression in TME of lung cancer,^35^ this can also be an adverse prognostic factor in lung cancer.^36^, ^37^ Neutrophils have also been revealed to facilitate the metastatic spread of cancer.^38^ A higher fraction of resting mast cells has been verified to be associated with longer OS among NSCLC patients.^39^ Additionally, we further explored the immune checkpoints and uncovered a high expression of immunosuppressive receptors (CTLA4, PD‐1, LAG3, BTLA, and TIGIT) and immunosuppressive ligands (PD‐L1, PD‐L2 and TNFSF14) in the high‐risk cohort. Overexpression of these molecules might facilitate tumor immune escape in LUAD and further deteriorate the prognosis of patients. The findings of immune checkpoints in the present study could also help identify potential immunotherapy targets and develop individual treatment strategies for LUAD patients.

Our study had a few limitations. First, this was retrospective research based on the gene expression profile and a few clinical factors from the TCGA database, and some specific clinical information related to lung cancer may have been unavailable. Second, a single data source was only applied in the present study, and the findings await further validation in other datasets, cell lines and tissue samples. Third, the prognostic value and biological functions of ARGs have not yet been fully elucidated in this study.

In summary, a novel anoikis‐related signature was identified for LUAD patients in our study. The ARGs in the signature could refine the prediction performance of LUAD survival outcome and evaluate the immune conditions and forecast the immune checkpoints for LUAD patients. The ARGs identified in the present study may offer potential therapeutic targets or prognostic predictors for LUAD patients.

## AUTHOR CONTRIBUTIONS

SQL had full access to all the data in the study and take responsibility for the integrity of the data and the accuracy of the data analysis. Study concept and design: SQL and XYD. Acquisition and analysis of data: XYD and GC. Drafting the manuscript: XYD. Proofread the manuscript for important intellectual content: all authors. Approval of the final version manuscript: all authors. Accountable for all aspects of the work: all authors.

## CONFLICT OF INTEREST

The authors declare that they have no conflicts of interests.

## Supporting information


**Table S1**. Anoikis‐related genes

## Data Availability

All data can be retrieved from the TCGA database (https://portal.gdc.cancer.gov).
