# Supplementary material for: Identification of a novel anoikis‐related gene signature to predict prognosis and tumor microenvironment in lung adenocarcinoma
Source: Thorac Cancer. 2022 Dec 11;14(3):320–30. doi: 10.1111/1759-7714.14766 (PMC9870742; doi:10.1111/1759-7714.14766)
Supplement: Supplementary file 1 — Table S1. Anoikis‐related genes [file TCA-14-320-s001.docx]

**Table S1. Anoikis-related genes**

BRMS1

PTK2

NTRK2

BCL2L11

SRC

CEACAM6

CAV1

AKT1

ITGB1

CEACAM5

EGFR

BCL2

CASP8

SIK1

PTRH2

STAT3

TLE1

DAPK2

CTNNB1

ZNF304

MAPK1

BMF

ITGA5

TP53

MCL1

BCL2L1

CASP3

CDH1

BAD

PIK3CA

PAK1

ITGAV

FN1

MAPK3

PTGS2

BAX

BCAR1

PTEN

ERBB2

ANGPTL4

PDK4

CYCS

BRAF

YAP1

ANKRD13C

ITGA2

ANXA5

BIRC5

MTOR

TIMP1

BDNF

CSPG4

BSG

AKT2

STK11

IGF1

IGF1R

ITGA6

ILK

CFLAR

RHOA

HIF1A

DAP3

MYBBP1A

TLE5

ITGA3

PTK2B

CCND1

CTTN

CALR

ATF4

CDCP1

PLAUR

SKP2

CHEK2

HGF

E2F1

EGF

PIK3CG

ITGB4

DAPK1

MAPK8

PIK3R1

PIK3R3

MAP2K1

CXCL12

LGALS3

FBXW7-AS1

BAK1

ABHD4

CD44

ITGA4

FADD

PHLDA2

TGFB1

HMCN1

MMP2

CEBPB

CEMIP

CDKN3

CBL

CASP9

SFN

MTDH

PRKCA

TNFRSF10B

CXCL8

MIR200C

AR

CDKN2A

CPT1A

PIK3CB

CLDN1

MIR204

MIR26A1

CDKN1A

CDKN1B

KLF12

NTRK1

PLAU

MYC

SMAD4

PLK1

MUC1

LGALS1

PYCARD

SESN2

ITGB3

KRAS

THBS1

BID

HRAS

CDK11B

CDK11A

XIAP

PPARG

IL6

MIR145

CCR7

MSLN

RAC1

GRHL2

BIRC3

NOTCH1

RHOG

CCAR2

NQO1

MMP13

FAS

MTA1

MYO5A

EDA2R

CCN6

MMP9

ABL1

MAPK11

SOD2

PTHLH

PDGFB

GLI2

EZH2

RIPK1

CXCR4

HMGA1

SIK2

TNFSF10

ANGPTL2

S100A4

NTF3

ETV4

MIR21

MIR124-1

HTRA1

LATS1

CEACAM3

EIF2AK3

LAMC2

LAMA3

LAMB3

CDH2

CSNK2A1

EDIL3

ZEB2

TLN1

EPHA2

SIRT3

OLFM3

CLU

SPINK1

CPEB2

NAT1

TSG101

MIR200A

MIR6744

SERPINA1

AKT3

RELA

TNFRSF1A

FASLG

AFP

ITGA8

NOX4

PBK

SATB1

CD63

EEF1A1

LTB4R2

MAVS

HRC

CCN2

RHOB

PPP1R13B

PLG

MET

RAF1

PARP1

PRKCQ

BRCA2

RB1

SP1

HAVCR2

DOCK1

VTN

INHBB

PDCD4

PRPF4B

RANBP9

SESN1

SESN3

CD24

ZBTB7A

MIR141

ELANE

KDR

MDM2

NFE2L2

ZEB1

KL

PRKCI

CRYAB

EPHB6

FGF2

HK2

LTF

IQGAP1

MGAT5

SDCBP

ABHD2

SPIB

TRIM31

MIR1827

PDGFRB

PLAT

TLR3

NRAS

ROCK1

PAK4

VEGFA

CASP10

PIN1

IL1RAP

UBE2C

YWHAZ

TWIST1

BMP6

BNIP3L

ELK1

KDM3A

PRDX4

BNIP3

LMO3

ZNF32

MIR200B

MIR525

MIR363

TUBB3

HSP90B1

SLC2A1

HMOX1

PTPN11

PRKACA

PAK3

CD36

PIK3R2

PPP2CA

CASP6

CDH3

EEF2K

LRP1

PAK2

PTK6

LPAR1

TCF7L2

CEACAM1

GDF2

GLO1

IL17A

RBL2

SIRPA

TRAF2

ADCY10

VPS37A

TNFRSF12A

APOBEC3G

BAG1

COL13A1

MNX1

RAD9A

IFI27

MEGF11

ITPRIP

BCL2L15

SNAI2

PTPN1

NOTCH3

GLUD1

SIRT1

FASN

MYH9

RPS6KB1

TPM1

PPP2R1A

COL4A2

CTNND1

CD151

MMP11

ARHGEF7

PPP2R2A

SEMA7A

PPP2R5A

BST2

CCN1

PPP2R2D

CCDC178

MIR10A

MIR30B

MIR30C1

SHC1
